# Adverse childhood experiences and cognitive function among older adults in low‐ and middle‐income countries: A systematic review and meta‐analysis

**DOI:** 10.1002/alz70860_100735

**Published:** 2025-12-23

**Authors:** Che Henry Ngwa, Sawsan Abdulrahim, Meiya Saad, Mayssan Kabalan, Maria Rita Lteif, Yara Yazbek, Rim Nehme, Monique Chaaya, Martine Elbejjani

**Affiliations:** ^1^ Faculty of Health Sciences, American University of Beirut, Beirut, Beirut, Lebanon; ^2^ Faculty of Health Sciences, American University of Beirut, Lebanon, Beirut, Lebanon; ^3^ Faculty of Medicine, American University of Beirut, Beirut, Lebanon

## Abstract

**Background:**

A large body of evidence suggests that adverse childhood experiences (ACEs) are associated with poorer cognitive functioning and cognitive impairment later in life. Yet, most of the evidence comes from high‐income countries, which often have greater social and healthcare resources and infrastructure to mitigate the effects of ACEs. We performed a systematic review and meta‐analysis of studies that examined the association between ACEs and cognitive function/impairment among adults in low and middle‐income countries (LMICs). We also conducted a narrative synthesis of whether and how ACE characteristics, such as frequency, severity, and age at exposure, were included in studies.

**Method:**

We searched four electronic databases from conception until August 2024 for studies investigating the association between individual ACEs and cognitive function and cognitive impairment among older adults in LMICs. Two authors independently screened and performed data extraction and quality assessment for each study. We calculated the pooled estimate using a random‐effects model. We also conducted a sub‐meta‐analysis using studies reporting on the same ACE.

**Result:**

Our search yielded 10883 records, of which 13 articles were included in the final meta‐analysis. The most examined ACEs include emotional neglect, parental death, and childhood illness. The pooled effect of ACE on cognitive function was β = ‐0.27 (95% CI ‐0.37, ‐0.16), and on cognitive impairment was OR = 1.12 (95% CI 0.95, 1.32). In sub‐meta‐analyses, parental death (β = ‐0.37; 95% CI ‐0.52, ‐0.22) and emotional neglect (β = ‐0.41; 95% CI ‐0.75, ‐0.08) were associated with poorer cognitive function. We found considerable heterogeneity (I^2^>75%) in the meta‐analysis for both outcomes. In most studies, ACEs were measured crudely without considering the frequency, severity, or age at exposure.

**Conclusion:**

ACEs are associated with an increased risk of poor cognitive function and impairment in old age in LMICs, consistent with findings from high‐income countries. However, the evidence from LMICs is scanty, with most of the included studies conducted in China. Consideration of key ACE characteristics like frequency, severity and age at exposure remain largely unexamined despite their potential importance. More research in other LMICs considering contextual ACEs is needed to build the evidence base.

